# Macrophages Modulate Hepatic Injury Involving NLRP3 Inflammasome: The Example of Efavirenz

**DOI:** 10.3390/biomedicines10010109

**Published:** 2022-01-05

**Authors:** Fernando Alegre, Alberto Martí-Rodrigo, Miriam Polo, Dolores Ortiz-Masiá, Celia Bañuls, Marcello Pinti, Ángeles Álvarez, Nadezda Apostolova, Juan V. Esplugues, Ana Blas-García

**Affiliations:** 1 Departamento de Farmacología, Facultad de Medicina, Universidad de Valencia, 46010 Valencia, Spain; fernando.alegre@uv.es (F.A.); alberto.marti-rodrigo@uv.es (A.M.-R.); miriam.polo@uv.es (M.P.); nadezda.apostolova@uv.es (N.A.); juan.v.esplugues@uv.es (J.V.E.); 2 Servicio de Endocrinología, FISABIO-Hospital Universitario Dr. Peset, 46017 Valencia, Spain; celia.banuls@uv.es (C.B.); ana.blas@uv.es (A.B.-G.); 3 Departamento de Medicina, Facultad de Medicina, Universidad de Valencia, 46010 Valencia, Spain; m.dolores.ortiz@uv.es; 4 Centro de Investigación Biomédica en Red Enfermedades Hepáticas y Digestivas (CIBERehd), 46010 Valencia, Spain; 5 Department of Life Sciences, University of Modena and Reggio Emilia, 41121 Modena, Italy; marcello.pinti@unimore.it; 6 Departamento de Fisiología, Universidad de Valencia, 46010 Valencia, Spain

**Keywords:** antiviral therapy, inflammation, fibrogenesis, DILI, macrophage polarization

## Abstract

Drug-induced liver injury (DILI) constitutes a clinical challenge due to the incomplete characterization of the mechanisms involved and potential risk factors. Efavirenz, an anti-HIV drug, induces deleterious actions in hepatocytes that could underlie induction of the NLRP3 inflammasome, an important regulator of inflammatory responses during liver injury. We assessed the potential of efavirenz to modulate the inflammatory and fibrogenic responses of major liver cell types involved in DILI. The effects of efavirenz were evaluated both in vitro and in vivo. Efavirenz triggered inflammation in hepatocytes, in a process that involved NF-κB and the NLRP3 inflammasome, and activated hepatic stellate cells (HSCs), thereby enhancing expression of inflammatory and fibrogenic markers. The NLRP3 inflammasome was not altered in efavirenz-treated macrophages, but these cells polarized towards the anti-inflammatory M2 phenotype and displayed upregulated anti-inflammatory mediators. Conversely, no evidence of damage was observed in efavirenz-treated animals, except when macrophages were depleted, which resulted in the in vivo manifestation of the deleterious effects detected in hepatocytes and HSCs. Efavirenz elicits a cell-specific activation of the NLRP3 inflammasome in hepatocytes and HSCs, but macrophages appear to counteract efavirenz-induced liver injury. Our results highlight the dynamic nature of the interaction among liver cell populations and emphasize the potential of targeting macrophage polarization as a strategy to treat NLRP3 inflammasome-induced liver injury.

## 1. Introduction

Dysregulated inflammatory responses contribute to the pathogenesis of most acute and chronic liver diseases, including drug-induced liver injury (DILI), which constitutes a clinical challenge due to the difficulties to characterize the mechanisms involved and the risk factors responsible for its worsening [1]. Excessive inflammation is crucial in drug-induced hepatic damage and, when associated with hepatotoxic insults, it can lead to fibrosis [2]. Hepatic inflammation and fibrogenesis are mediated by the inflammatory pathway regulated by NF-κB and by inflammasomes, intracellular complexes that recognize pathogen- and damage-associated molecular patterns (PAMPs and DAMPs) [3,4]. NLRP3—whose domains include the NOD-like receptor protein 3 (NLRP3), adaptor molecule ASC (also referred to as PYCARD) and serine protease Caspase-1—is the inflammasome most commonly activated in liver diseases [5,6]. Upon detecting noxious patterns, Caspase-1 is cleaved and activated, thus inducing interleukin-1β (IL-1β) and interleukin-18 (IL-18) maturation. NLRP3 inflammasome is triggered in response to diverse stimuli that frequently involve the production of reactive oxygen species (ROS) and calcium mobilization [3]. We have previously described how efavirenz (EFV), a widely used antiretroviral drug in the treatment of human immunodeficiency virus (HIV) infection, alters the expression of some inflammation-related genes [7] and exhibits a pattern of actions in hepatocytes—it increases ROS levels and disrupts calcium homeostasis following mitochondrial dysfunction, cell survival-promoting autophagy and endoplasmic reticulum (ER) stress [8,9,10,11]—that could underlie induction of the NLRP3 inflammasome and hepatic inflammation. Fibrogenesis is a multicellular response linked closely to inflammatory signaling pathways, during which activated hepatic stellate cells (HSCs) interact with liver-resident and immune cells [12] and trigger associated processes that exacerbate inflammation [13]. After initiation and perpetuation of liver injury, regression/resolution is mediated by different mechanisms, including HSC deactivation and induction of the immune system. Specifically, hepatic macrophages are central players in liver homeostasis and pathogenesis, with complex roles in both inflammation and resolution of injury [14]. Importantly, some of the pathophysiological processes involved in the activation of HSCs have been attributed to EFV treatment [15,16], while HIV has been reported to directly induce cytokine secretion and collagen production in HSCs [17]. As this non-nucleoside reverse transcriptase inhibitor (NNRTI) increases the risk of dyslipidemia, hepatic steatosis and accumulation of lipotoxic lipids [18,19], clarification of its actions on liver function is essential to choose the best antiretroviral treatment in certain clinical situations. In fact, increasing evidence suggests beneficial effects after switching from EFV-containing therapies to regimens containing newer antiretroviral drugs [19,20,21]. The present study assessed the effects of EFV, a proven pharmacological model of mitochondrial dysfunction and ER stress, on the regulation of the inflammatory and fibrogenic response of key hepatic cell types (hepatocytes, HSCs and macrophages), focusing on the interactions between these cells and the role of this drug in NF-κB and NLRP3 inflammasome activation. 

## 2. Materials and Methods

### 2.1. Reagents and Drugs

Cell culture reagents from Gibco (Life Technologies, Madison, WI, USA); EFV from Sequoia Research Products (Pangbourne, UK), dissolved in methanol and used at clinically relevant concentrations; fluorochromes from Molecular Probes (Life Technologies, Madison, WI, USA), except Hoechst 33342, from Sigma-Aldrich (Steinheim, Germany). Unless stated otherwise, all other reagents were from Sigma-Aldrich.

### 2.2. Cell Culture and Treatments 

Cell lines and primary human cells were cultured and treated (24 h) as described in the Supplementary Material.

### 2.3. In Vivo Procedures

Female C57BL/6 mice (10 weeks old) were supplied by Janvier Labs. (Le Genest Saint Isle, France). Animal dosages (p.o.) were calculated according to the maximum daily therapeutic dose of EFV for humans (600 mg) using the Food and Drug Administration’s normalization method based on body surface area [22]. A total of 24 h after administration, mice were sacrificed by cervical dislocation, and liver samples were processed to isolate RNA. Key experiments were performed with gadolinium chloride (GdCl_3_, Fisher Scientific Acros, Ottawa, ON, Canada), a selective inhibitor of macrophages, administered 24 h before EFV (20 mg/kg in saline, i.v.) [23]. Animal procedures were in accordance with the University of Valencia’s guidelines for the care and use of laboratory animals and approved by the local ethics committee.

### 2.4. Protein Extraction and Western Blot (WB) Analysis

Total cell and nuclear protein extracts were employed to analyze the expression of different proteins (Supplementary Material; primary antibodies listed in Supplementary Table S1).

### 2.5. Electrophoretic Mobility Shift Assay (EMSA)

Nuclear extracts from Hep3B cells were used to examine NF-κB binding to a potential binding site in the *SERPINE1* promoter. Analysis of the *SERPINE1* gene revealed a potential binding site (consensus sequence) for NF-κB in its promoter (from −368 to −354 positions relative to the transcription start site) and, consequently, EMSA was performed to examine NF-κB binding to the aforementioned sequence. Nuclear extracts from Hep3B cells (5 × 10^6^ cells) were obtained as described above. Synthetic oligonucleotides (biotin-labeled) were synthetized (TIB MOLBIOL GmbH Berlin, Germany) and used as probes (5′-BIOTEG-GATCTAGGGCAGTTCCAGG 3′). A mutated probe-binding site (*SERPINE1* mt: 5′-BIOTEG-GATCTAGGGCAGGGGGAGG3′) or excess unlabeled probes (XS) were used as controls. Nuclear extracts (10 µg) were incubated for 5 min, with or without an excess unlabeled probe, in DNA binding buffer (Pierce, Rockford, IL, USA) supplemented with 5 mM MgCl_2_, 50 ng/µL poly-d[I-C] (Pierce, Rockford, IL, USA), 0.05% NP40 (Pierce, Rockford, IL, USA) and 2.5% glycerol at room temperature. The labelled probe (25 fmol) was then added to the reaction mixture and incubated for 30 min at room temperature in a final volume of 20 µL. DNA–protein was resolved in a 6% non-denaturing polyacrylamide gel, as described previously. DNA–protein complexes were transblotted to Biodyne^®^ B nylon membrane (Pierce, Rockford, IL, USA), probed with streptavidin-horseradish peroxidase conjugate (Pierce, Rockford, IL, USA) and developed by enhanced chemiluminescence. Some nuclear extracts from EFV-treated cells were also incubated with a primary antibody anti-NF-κB (p65 subunit, 1:250, Invitrogen, Life Technologies, Madison, WI, USA) prior to EMSA to perform a supershift assay to assess the formation of complexes NF-κB-DNA-antibody that have less mobility.

### 2.6. RNA Isolation and Real Time RT-PCR

Total mRNA was isolated using an RNeasy Mini Kit (Qiagen, Hilden, Germany). A total of 1 µg of total RNA was employed to synthetize cDNA with the PrimeScript RT Reagent Kit (Takara Biotechnology, Shiga, Japan). Real-time PCR reactions were performed by mixing 1 µL of cDNA with the SYBR^®^ Premix Ex Taq™ (Takara Biotechnology, Shiga, Japan) in a Lightcycler^®^ 96 Real-Time PCR System (Roche Diagnostics, Mannheim, Germany). Sequences of human and murine primers are shown in Supplementary Table S2; *β-actin* expression was used as an internal control. The threshold cycle (C_T_) was determined and relative gene expression was expressed as follows: change in expression (fold) = 2^−Δ(ΔC^_T_^)^ where ΔC_T_ = C_T_ (target gene) − C_T_ (housekeeping gene), and Δ(ΔCT) = ΔC_T_ (treated) − ΔC_T_ (control).

### 2.7. Real Time PCR Assays

Analysis of the relative expression of different genes involved in cellular stress and inflammation was performed in LX2 cells and macrophages using a pre-validated set of real time PCR assays directly dried in wells (PrimePCR assays Bio-Rad Laboratories, Hercules, CA, USA), following the manufacturer’s instructions. The list of the genes evaluated and additional details are in Supplementary Material.

### 2.8. Fluorescence Microscopy and Static Fluorescence

Cell proliferation/survival, mitochondrial membrane potential, mass and superoxide production, ER signal, lysosome signal and intracellular lipid accumulation were analyzed using specific fluorochromes with an IX81 Olympus fluorescence microscope with “ScanR” static cytometry software version 2.03.2 (Olympus, Hamburg, Germany). Details are provided as Supplementary Material. 

### 2.9. Confocal Microscopy

LX2 cells were cultured on chambered borosilicate coverglasses (Thermo Scientific, Rockford, IL, USA), treated, and stained with specific fluorochromes to assess colocalization between LC3 and lipid droplets. Following treatments, cells were fixed for 15 min with 100% methanol (VWR Chemicals, Radnor, PA, USA) at −20 °C and were then blocked for 1 h at room temperature in PBS containing 5% goat serum and 0.3% Triton™ X-100 (Sigma Aldrich, Steinheim, Germany). Cells were incubated for 18 h in PBS with 1% BSA and 0.3% Triton X-100 containing LC3 XP Rabbit mAb antibody (4599, Cell Signaling Technology, Danvers, MA, USA, 1:400) at 4 °C and washed with PBS. Samples were then incubated for 1 h with a secondary antibody conjugated with Alexa 488 Goat Anti-rabbit (A-11008, 1:500, Molecular Probes, Life Technologies, Madison, WI, USA) at room temperature, and Hoechst 33342 (3 µM, Sigma Aldrich, Steinheim, Germany) was added for the last 30 min. After washing cells with PBS, they were incubated for 10 min in HBSS containing 0.5 µM Nile Red. Fluorescence was detected with a Leica fluorescence confocal microscope (Leica microsystems CMS GmbH, Mannheim, Germany), and the Colocalization Colormap Plugin was used to calculate the Correlation Index (Icorr).

### 2.10. ELISA Assay

IL-1β concentration in cell supernatants (from treated LX2 cells) was determined using a commercially available enzyme-linked immunosorbent assay (ELISA) kit (Diaclone, Besançon Cedex, France) according to the manufacturer’s instructions. Absorbance was measured at 450 nm with an Infinite 200 PRO series spectrophotometer (TECAN Trading AG, Männendorf, Switzerland).

### 2.11. Caspase-1 Activity Assay

A colorimetric assay kit (Biovision Inc., Milpitas, CA, USA) was employed to measure this parameter in Hep3B and LX2 cells following the manufacturer’s instructions, with slight modifications. In brief, protein lysates were obtained with Cell Lysis Buffer and protein concentration was determined with the BCA protein assay kit. For equal amounts of protein in each sample (400 µg), YVAD-pNA was added in a 96-well plate. This substrate is cleaved by the active Caspase-1, and subsequently releases the chromophore pNA, whose absorbance we measured at 405 nm in an Infinite^®^ 200 PRO spectrophotometer (TECAN, Männedorf, Switzerland). Fold increase in Caspase-activity was determined by comparison with untreated control.

### 2.12. Immunohistochemistry

Immunostaining for F4/80 (AbD Serotec, Bio-Rad Laboratories, Hercules, CA, USA, 1:150) was performed in 5 µM paraffin-embedded liver samples. A horse anti-mouse/rabbit biotinylated antibody (Vector Laboratories, Burlingame, CA, USA; 1:200) was used as a secondary antibody. The Vectastain elite ABC system kit (Vector Laboratories, Burlingame, CA, USA) was employed for signal development. All tissues were counterstained with hematoxylin, and the specificity of the immunostaining was confirmed by the absence of signal when primary or secondary antibodies were omitted.

### 2.13. MPO Activity

Liver MPO activity was determined spectrophotometrically in mice liver homogenates by measuring absorbance at 450 nm. Frozen liver samples were homogenized in pH 4.7 buffer (0.1 M NaCl, 0.02 M Na_2_HPO_4_, 0.015 M Na_4_EDTA; 1 g tissue/19 mL buffer) using a MACS Dissociator and centrifuged (300× *g* for 10 min). Pellets underwent hypotonic lysis (0.2% NaCl and later NaCl 1.6% and glucose 5%; both at 1 g tissue/15 mL buffer). After centrifugation (300× *g* for 10 min), pellets were resuspended in pH 5.4 0.05 M Na_2_HPO_4_ buffer containing 0.5% hexadecyltrimethylammonium bromide (HTAB) and homogenized. These extracts underwent three freeze–thaw cycles using liquid nitrogen and were centrifuged once again (10,000× *g* for 15 min). Finally, pellets were resuspended in 1 mL of pH 5.4 buffer and 50 mL of tetramethylbenzidine (1.6 mM), after which hydrogen peroxide (0.052%) was added. Following 15 min incubation (37 °C, in darkness), absorbance was measured at 450 nm using an Infinite^®^ 200 PRO spectrophotometer (TECAN, Männedorf, Switzerland). The results are expressed as a percentage of MPO abs/mg of tissue.

### 2.14. Data and Statistical Analyses

All images display representative data for at least 3 independent experiments. Data (mean ± SEM) were analyzed using GraphPad Prism v.5 with a one-way ANOVA followed by a Newman–Keuls test or a *t*-test. Rotenone, thapsigargin and C.LPS were analyzed separately with a *t*-test. In most cases, data are represented as a percentage of the control, with the negative control (untreated cells) considered 100%.

## 3. Results

### 3.1. Effects of EFV on Inflammatory Pathways in Hep3B Cells

#### 3.1.1. Activation of the NF-κB Signal Pathway

**Figure 1:**
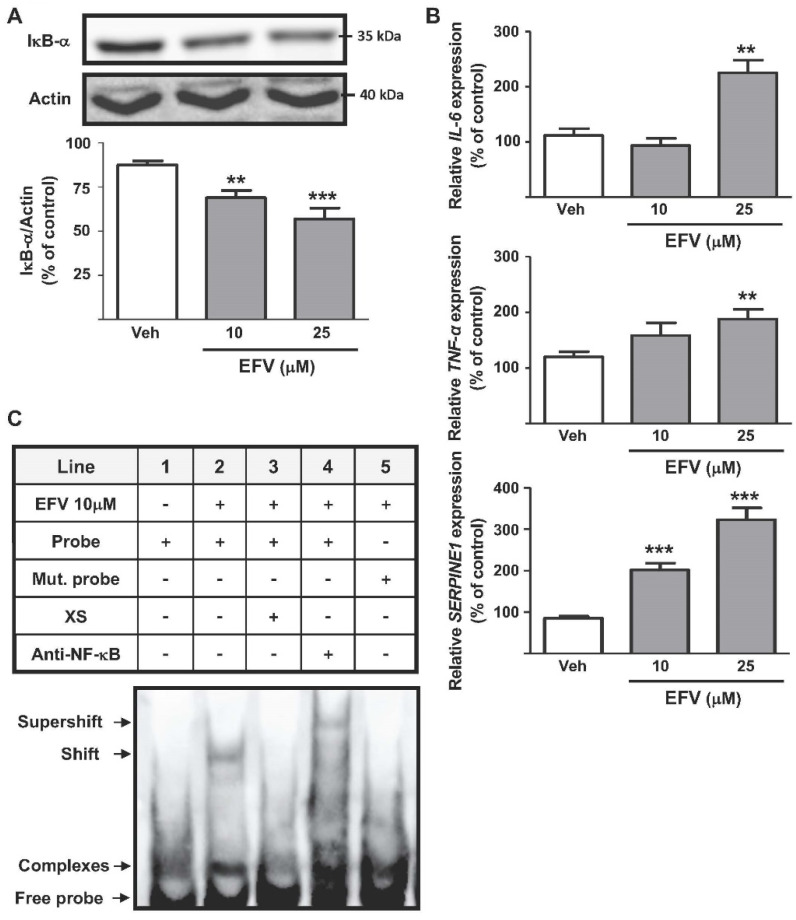
Efavirenz triggers the NF-κB pathway in Hep3B cells. (**A**) Representative Western Blot image of IκB-*α* and summary of densitometry data after 24 h treatment (n = 8). (**B**) Relative mRNA expression levels of the pro-inflammatory cytokines *IL-6* and *TNF-α* (RT-PCR). Data were normalized versus the housekeeping gene *β-actin* (n = 5). (**C**) EMSA assay showing the binding of NF-κB (subunit p65) to the promoter of *SERPINE1* in EFV-treated cells. Representative image of the different conditions detailed in the table (n = 5). Data (mean ± SEM) were calculated as a percentage of control (untreated cells) and analyzed by a one-way ANOVA multiple comparison test followed by a Newman–Keuls test (** *p* < 0.01, *** *p* < 0.001 versus the respective solvent).

EFV enhanced the translocation of NF-κB to the nucleus, shown by the concentration-dependent decrease in protein levels of the NF-κB inhibitor IκB-*α* (Figure 1A) and an increase in the levels of the p65 subunit in nuclear extracts (data not shown). Additionally, EFV upregulated the expression of different inflammation-related genes associated with this transcription factor, specifically that of *TNF-α*, *IL-6* and *SERPINE1* (Figure 1B). An EMSA assay demonstrated that EFV induced both the nuclear accumulation of NF-κB and its specific binding to the consensus sequence located within the *SERPINE1* promoter, thereby upregulating the transcription of this pro-fibrotic gene (Figure 1C). Further confirmation of NF-κB interaction with the consensus sequence of *SERPINE1* was obtained with a supershift assay (line 4). To assess the specificity of the experiment, EFV (10 µM) -treated extracts were incubated with excess unlabeled primer or mutated probe, which resulted in complete loss of the signal (lines 3 and 5, respectively).

#### 3.1.2. Activation of the NLRP3 Inflammasome

**Figure 2:**
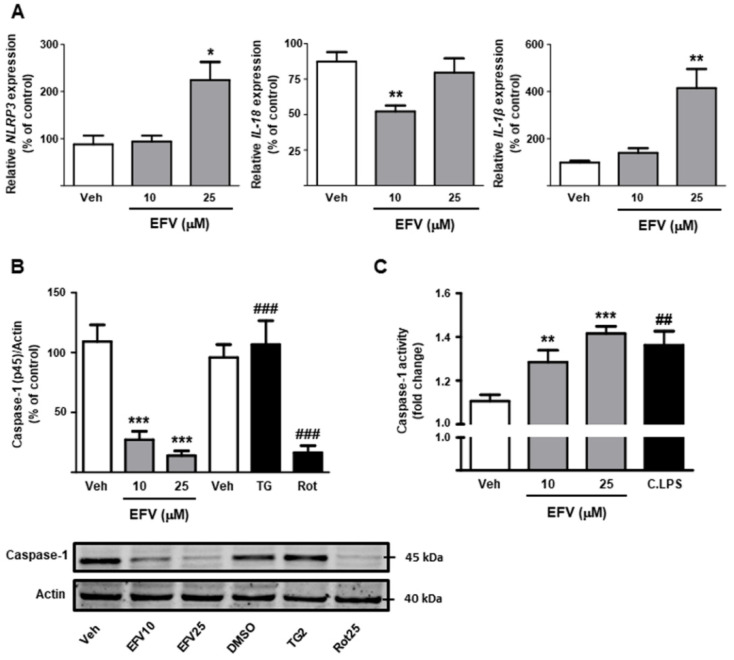
Efavirenz activates NLRP3 inflammasome components in Hep3B cells. (**A**) Determination of relative mRNA expression levels of genes associated with the NLRP3 inflammasome (*NLRP3*, *IL-18* and *IL-1β*) by RT-PCR. Data were normalized versus the housekeeping gene *β-actin* (n = 5–6). (**B**) Representative Western Blot image of the inactive form of Caspase-1 (45 kDa) and summary of densitometry data after 24 h treatment (n = 5). (**C**) Analysis of Caspase-1 activity using a colorimetric assay. All data (represented as mean ± SEM, n = 5–6) were calculated as a percentage of control (untreated cells) and analyzed by one-way ANOVA multiple comparison test followed by a Newman–Keuls test (* *p* < 0.05, ** *p* < 0.01, *** *p* < 0.001 versus the respective solvent). Thapsigargin (TG), rotenone (Rot) and cocktail of LPS (C.LPS) were independently analyzed by a Student’s *t*-test (^##^ *p* < 0.01, ^###^ *p* < 0.001).

EFV significantly enhanced the transcription of *NLRP3* and that of the effector cytokine *IL-1β*, whereas expression of *IL-18* actually decreased (10 µM) (Figure 2A). Activation of the NLRP3 inflammasome was confirmed by a profound reduction in the protein expression of procaspase-1 in EFV-treated cells. Importantly, the inducer of mitochondrial dysfunction rotenone generated a similar decrease that was not reproduced by the classic ER stress-inducer thapsigargin (Figure 2B). Colorimetric assessment of the activity of Caspase-1 following EFV treatment revealed a significant and concentration-dependent increase that reached levels comparable to those induced by a cocktail of LPS (C.LPS) (Figure 2C).

### 3.2. Effects of EFV on Activation and Inflammatory Pathways in LX2 Cells

#### 3.2.1. Undermining of Cellular Function: Mitochondria and ER

**Figure 3:**
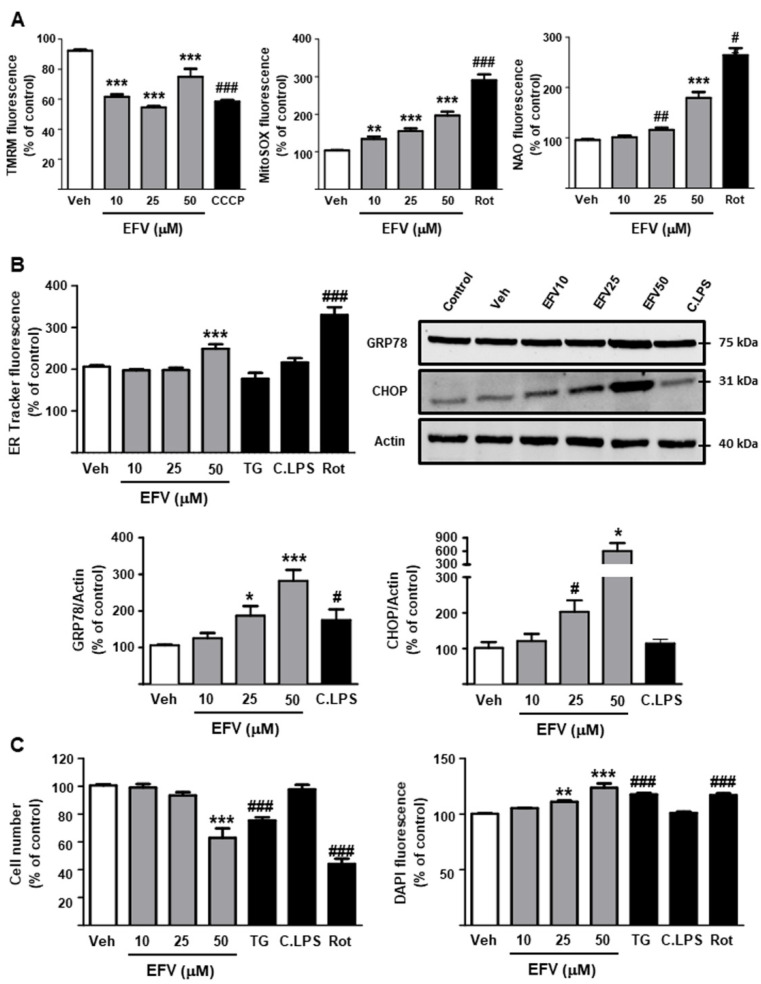
Efavirenz treatment alters cellular function in LX2 cells, thus promoting their activation. (**A**) Quantitative analysis of mitochondrial function by fluorescence microscopy: mitochondrial membrane potential (TMRM fluorescence), mitochondrial superoxide production (MitoSOX fluorescence), mitochondrial morphology and mass (NAO fluorescence) (n = 5). (**B**) Assessment of unfolded protein response and endoplasmic reticulum stress: quantitative analysis of the endoplasmic reticulum signal (ER-Tracker Red fluorescence) and protein expression of the classic markers of these processes, GRP78 and CHOP. Representative Western Blot image of these proteins and summary of densitometry data after 24 h treatment (n = 5–6). (**C**) Assessment of cellular proliferation/viability: quantitative analysis of cell number (Hoechst 33342-nuclei) and nuclear fluorescence intensity (n = 6). Data were represented as mean ± SEM, calculated as percentage of control (untreated cells) and analyzed by one-way ANOVA multiple comparison test followed by a Newman–Keuls test (* *p* < 0.05, ** *p* < 0.01, *** *p* < 0.001 versus the respective vehicle). Thapsigargin (TG), rotenone (Rot) and cocktail of LPS (C.LPS) were independently analyzed by a Student’s *t*-test (^#^ *p* < 0.05, ^##^ *p* < 0.01, ^###^ *p* < 0.001).

EFV induced mitochondrial dysfunction in a concentration-dependent fashion, decreasing ΔΨ*_m_* (TMRM fluorescence) and enhancing the production of ROS (MitoSOX fluorescence). EFV-treated cells also exhibited increased NAO fluorescence, an indicator of mitochondrial mass alteration, but only at the highest concentration (50 µM) (Figure 3A). However, the protein expression of two important mediators of the unfolded protein response and ER stress, GRP78 and CHOP, was significantly and concentration-dependently augmented. Nevertheless, an increase in ER Tracker Red fluorescence suggestive of alterations in ER appearance was observed only in cells treated with 50 µM EFV (Figure 3B). Following treatment, a cell count to define how these alterations affected the proliferation/survival of this line of HSCs revealed a significant reduction at the highest concentration (50 µM). However, a concentration-dependent increase in Hoechst 33342 fluorescence was also observed, pointing to changes in nuclear size or morphology that are commonly associated with nuclear condensation in response to cellular stress (Figure 3C).

Considered together, these results show that EFV induces a profile of alterations in the functioning of LX2 cells similar to that previously described in hepatocytes (8–11), although the former cell line seems to be more resistant to EFV-induced damage.

#### 3.2.2. Degradation of Intracellular Lipid Droplets by Autophagy

**Figure 4:**
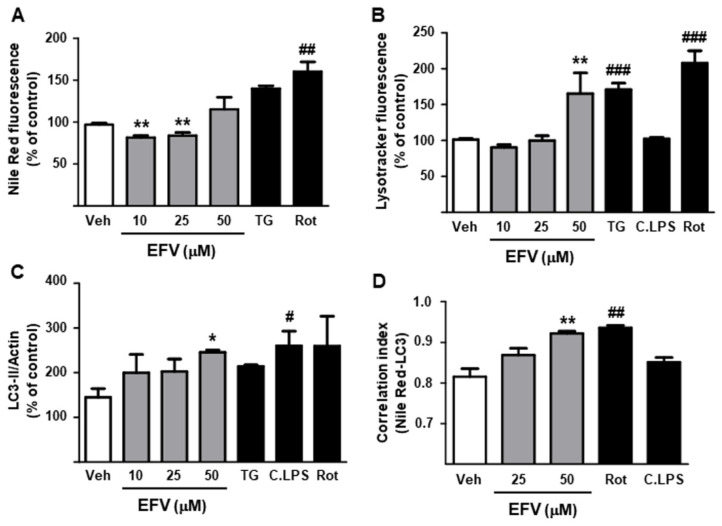

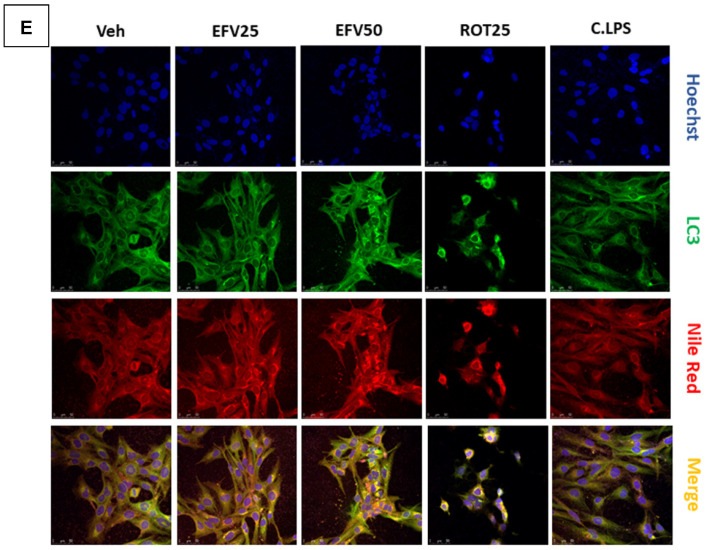
EFV-induced reduction in lipid droplets in LX2 cells is associated with activation of autophagy. (**A**) Intracellular content of lipid droplets evaluated by Nile Red fluorescence (n = 6). (**B**,**C**) Analysis of autophagy markers: lysosome signal (Lysotracker fluorescence) and protein expression of LC3-II (n = 5–6). (**D**) Correlation index between Nile Red and LC3 fluorescences (n = 5) (**E**) Representative images obtained from confocal microscopy experiments showing Hoechst 33342, LC3, Nile Red and merge (n = 5). All the data (represented as mean ± SEM) were calculated as a percentage of control (untreated cells) and analyzed by a one-way ANOVA multiple comparison test followed by a Newman–Keuls test (* *p* < 0.05, ** *p* < 0.01 versus the respective vehicle). Thapsigargin (TG), rotenone (Rot) and cocktail of LPS (C.LPS) were independently analyzed by a Student’s *t*-test (^#^ *p* < 0.05, ^##^ *p* < 0.01, ^###^ *p* < 0.001).

Autophagy is thought to drive HSC activation by providing essential energy substrates through hydrolysis of lipid droplets. Evaluation of intracellular lipid content revealed a differential response among EFV concentrations, with 10 and 25 µM diminishing the intensity of the Nile Red signal and 50 µM inducing a slight increase in this parameter (Figure 4A). Importantly, incubation with the autophagic inhibitor 3MA reversed this decrease in 25 µM EFV-treated cells, implicating autophagy in the effect of this NNRTI (data not shown). Analysis of Lysotracker Green fluorescence, an indicator of autophagic flux, showed a significant increase in intensity only with 50 µM (Figure 4B). Moreover, EFV induced changes in the expression of the autophagy-related protein LC3-II, also suggesting an induction of autophagy (Figure 4C). To ascertain if EFV was activating lipophagy in HSCs, we analyzed the correlation index between LC3 and Nile Red signal using confocal fluorescence microscopy. As shown in the representative images and in the analysis of the correlation index (Figure 4D), EFV altered the fluorescence of both signals, increasing Nile Red staining and favoring the accumulation of LC3-II-characteristic punctae, especially at high concentrations. It also induced a significant and concentration-dependent overlapping of green (LC3) and red signals (Nile Red), thereby pointing to the induction of an autophagic degradation of lipid droplets. Rotenone exerted similar effects to 50 µM EFV, whereas C.LPS altered neither the individual levels of LC3 or Nile Red nor the co-localization of their signals.

#### 3.2.3. Modulation of the Inflammatory Response in LX2 Cells

**Figure 5:**
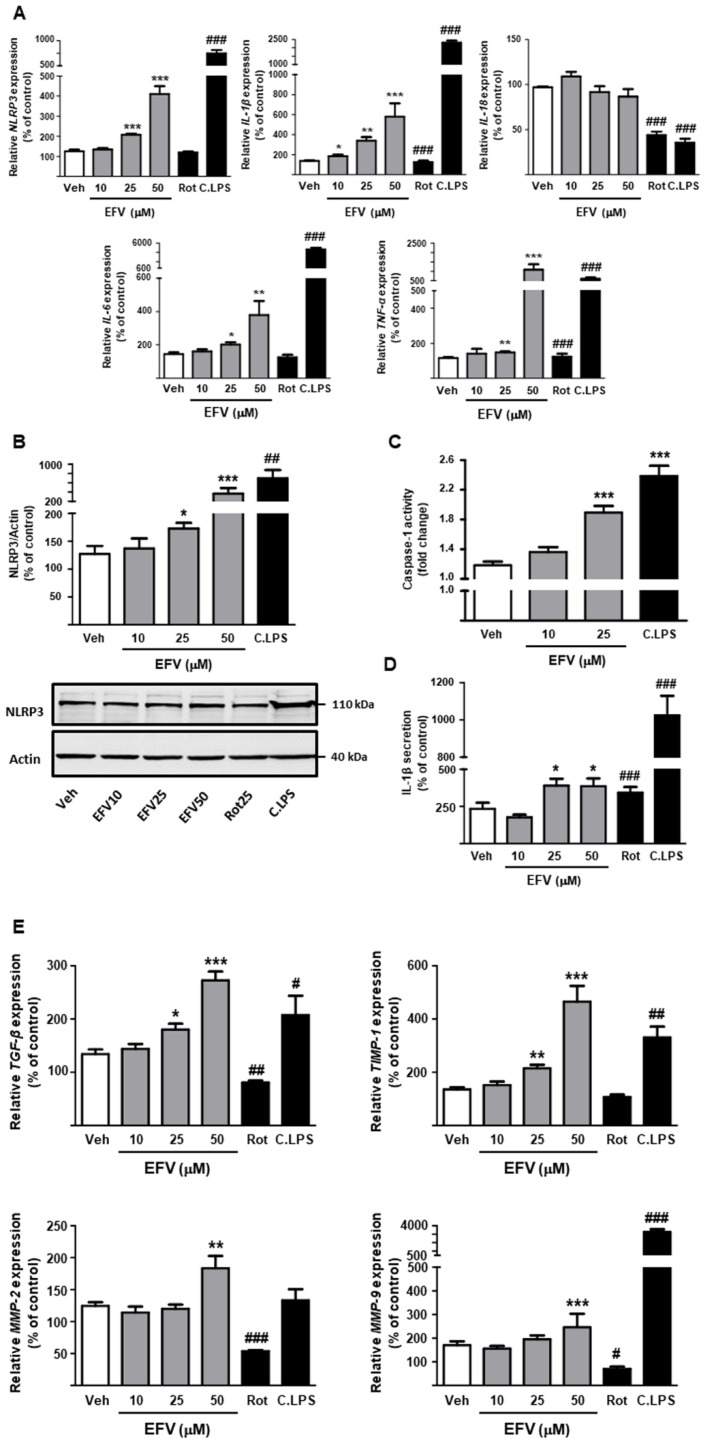
Efavirenz induces NLRP3 inflammasome-dependent pathways and upregulates fibrogenesis in LX2 cells. (**A**) Analysis of relative mRNA expression levels of inflammasome-related genes and pro-inflammatory cytokines by RT-PCR. Data were normalized versus the housekeeping gene *β-actin* (n = 5–6). (**B**) Representative Western Blot image of NLRP3 and summary of densitometry data obtained from independent experiments after 24 h treatment (n = 6). (**C**) Determination of Caspase-1 activity in total extracts of hepatic stellate cells (n = 7). (**D**) ELISA assay of IL-1*β* released from EFV-treated cells (n = 5). (**E**) Analysis of relative mRNA expression levels of different fibrogenic genes by RT-PCR. Data were normalized versus the housekeeping gene *β-actin* (n = 5–6). Data were represented as mean ± SEM, calculated as a percentage of control (untreated cells) and analyzed by a one-way ANOVA multiple comparison test followed by a Newman–Keuls test (* *p* < 0.05, ** *p* < 0.01, *** *p* < 0.001 versus the respective vehicle). Positive controls (rotenone -Rot- and cocktail of LPS -C.LPS-) were independently analyzed by a Student’s *t*-test (^#^ *p* < 0.05, ^##^ *p* < 0.01, ^###^ *p* < 0.001).

After determining the deleterious effects of this NNRTI on cellular function, we assessed whether these actions could lead to NLRP3 inflammasome activation and production of cytokines in HSCs. EFV concentration-dependently upregulated *NLRP3* expression in this cell line and induced overexpression of the pro-inflammatory cytokines *IL-1β*, *IL-6* and *TNF-α* (Figure 5A); as in Hep3B cells, no changes were observed in *IL-18* levels. The pro-inflammatory profile suggested by the RT-PCR results was confirmed by an array in which EFV enhanced the expression of these and other genes implicated in inflammation and cellular stress, such as *CASP1*, *IL-33*, *PYCARD* and *PANX1* (Supplementary Figure S1). As expected, WB of whole-cell protein extracts of EFV-treated cells revealed a significantly increased expression of NLRP3 (Figure 5B). Additional experimental approaches corroborated the activation of the NLRP3 inflammasome pathway, as LX2 cells incubated with 25 or 50 µM EFV displayed increased activity of Casp1 and enhanced secretion of IL-1*β* (Figure 5C,D). EFV-induced changes in NLRP3 inflammasome activation and inflammation were similar globally, but less severe than those elicited by C.LPS. Interestingly, and despite having shown similar effects to EFV in hepatocytes, rotenone did not affect these inflammatory parameters in HSCs.

#### 3.2.4. Activation of Fibrogenic Pathways

Our data indicated that EFV significantly upregulated the transcription of mediators of processes related to activated HSC, such as fibrogenesis (*TGF-β*), and altered matrix degradation (*TIMP-1*, *MMP-2* and *MMP-9*) (Figure 5E), although no significant alterations were observed in *COL1A1* expression (data not shown). This response, which was concentration-dependent, mimicked the one exerted by C.LPS, whereas rotenone-induced effects diverged. In fact, this pro-oxidant stimulus produced a downregulation of *TGF-β*, *MMP-2* and *MMP-9*, and no changes in *TIMP-1* expression.

### 3.3. Effects of EFV on Macrophage Function and Phenotype

**Figure 6:**
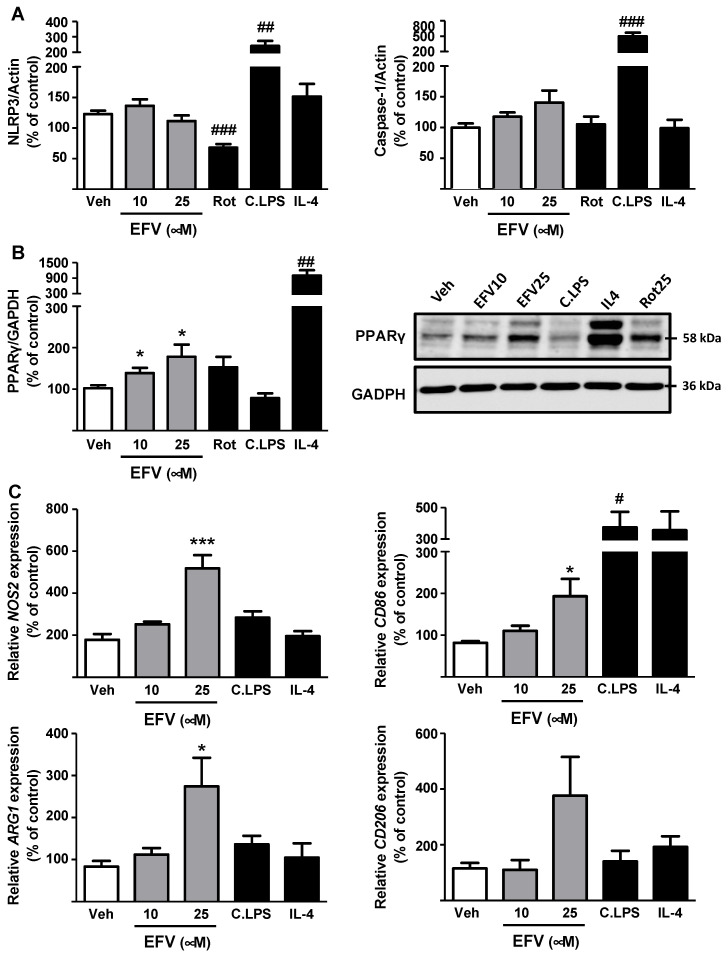
Efavirenz induces anti-inflammatory pathways in macrophages. (**A**) Determination of protein expression of NLRP3 and Caspase-1 (n = 8–10). (**B**) Representative Western Blot image of PPARγ and summary of densitometry data obtained from independent experiments after 24 h treatment (n = 5). (**C**) Analysis of relative mRNA expression levels of M1 (*NOS2* and *CD86*) and M2 markers (*ARG1 and CD206*) by RT-PCR. Data were normalized versus the housekeeping gene *β-actin* (n = 5–6). Data were represented as mean ± SEM, were calculated as a percentage of control (untreated cells) and analyzed by a one-way ANOVA multiple comparison test followed by a Newman–Keuls test (* *p* < 0.05, *** *p* < 0.001 versus the respective solvent). Positive controls employed (rotenone -Rot-, cocktail of LPS -C.LPS- and interleukin 4 -IL-4-,) were independently analyzed by a Student’s *t*-test (^#^ *p* < 0.05, ^##^ *p* < 0.01, ^###^ *p* < 0.001).

Array results demonstrated a decrease in *NLRP3* and *CASP1* expression in EFV-treated macrophages (24 h), while *PYCARD* and *IL-1β* remained slightly overexpressed. Regarding the P2XR7 pathway, we observed an enhanced expression of this receptor, although *PANX1* levels were similar to those in vehicle-treated cells. EFV also induced a significant and concentration-dependent upregulation of several anti-inflammatory mediators, including *TNFSF14*, *RIPK2*, and *NLRP12* (Supplementary Figure S2). NLRP3 and Caspase-1 protein levels were not altered, whereas WB showed a significant increase in PPARγ expression following incubation with EFV (Figure 6A,B). To assess the drug’s effect on macrophage polarization towards the M1/M2 subtypes we analyzed the expression of specific genes, observing that EFV upregulated the expression of both M1 (*NOS2* and *CD86*) and M2 markers (*ARG1* and *CD206*), thus promoting resolution of initial inflammation (Figure 6C). Overall, EFV’s actions painted an anti-inflammatory profile and differed from those induced by C.LPS.

### 3.4. EFV Induces Similar Results in Primary Hepatic Cells

**Figure 7:**
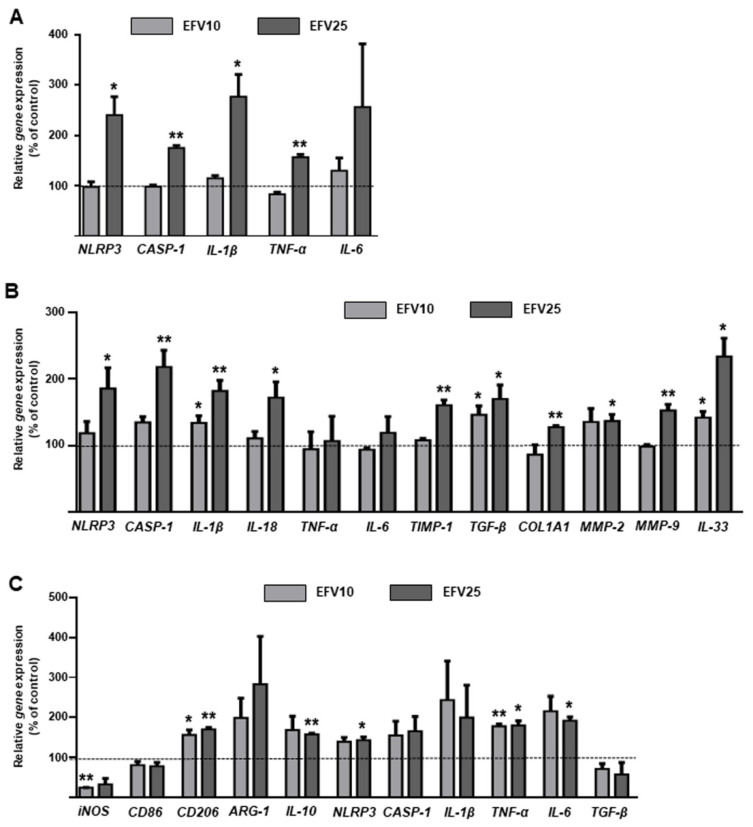
Effects of Efavirenz in human primary hepatocytes, hepatic stellate cells and Kupffer cells. Relative mRNA expression levels of inflammatory and fibrogenic genes in hepatocytes (**A**), hepatic stellate cells (**B**) and Kupffer cells (**C**) (RT-PCR). Data were normalized versus the housekeeping gene *β-actin* (n = 3). Data (mean ± SEM) were calculated as percentage of control (untreated cells) and analyzed by one-way ANOVA multiple comparison test followed by a Newman–Keuls test (* *p* < 0.05, ** *p* < 0.01 versus the respective solvent).

Human primary hepatocytes and HSCs exposed to EFV reproduced the same pattern of gene expression previously detected in human cell lines, but with greater intensity (Figure 7A,B). Results in Kupffer cells confirmed EFV-induced polarization towards the M2 phenotype, shown by a decrease in M1 markers and an increase in M2 markers (Figure 7C). These data validate the cellular models employed and suggest a dual role of EFV in the progression of liver injury.

### 3.5. Depletion of Kupffer Cells Modulates the Hepatic Effects of EFV In Vivo

**Figure 8:**
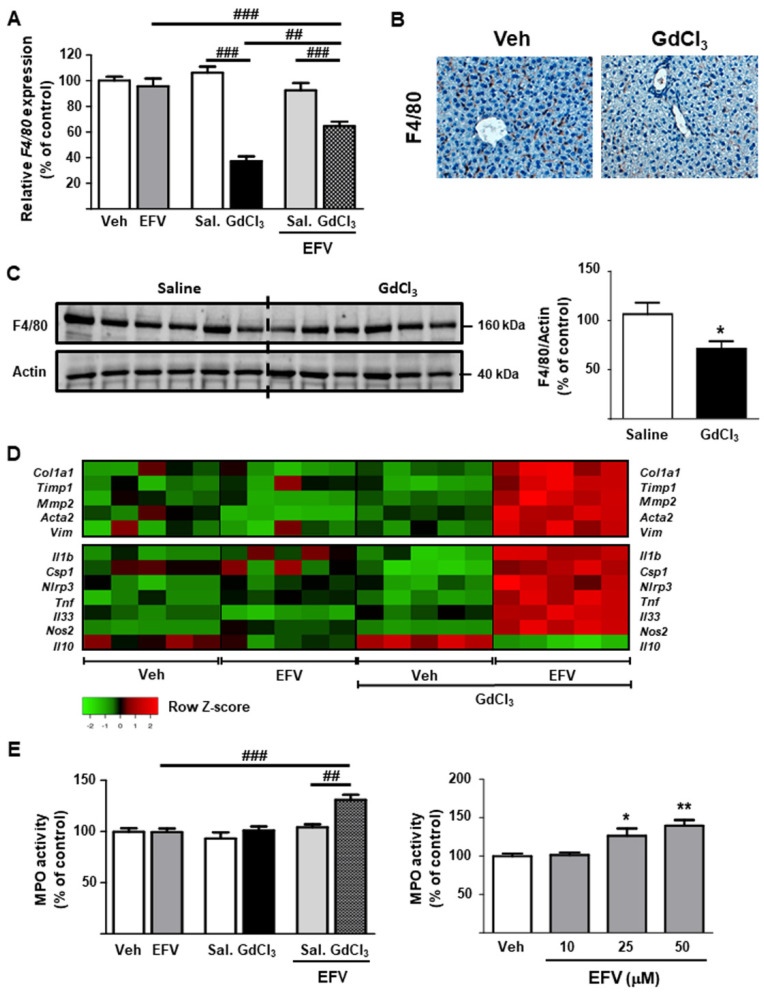
Depletion of Kupffer cells enhances Efavirenz-induced liver injury in vivo. (**A**–**C**) Confirmation of macrophage depletion in mice administered with gadolinium chloride (GdCl_3_): relative mRNA expression levels of *F4/80* (RT-PCR), representative images showing F4/80 immunostaining in murine livers (magnification 20×), and representative Western Blot image of F4/80 (each blot lane represents one mouse per condition—saline and GdCl_3_−) and summary of densitometry data (n = 6). (**D**) Heatmap representing color-coded expression levels of differentially expressed inflammatory and fibrogenic genes (assessed by RT-PCR). Data were normalized versus the housekeeping gene *β-actin* (n = 5). (**E**) Liver MPO activity (n = 5). Data (mean ± SEM) were analyzed by a one-way ANOVA multiple comparison test followed by a Newman–Keuls test (* *p* < 0.05, ** *p* < 0.01 versus vehicle) or by a Student’s *t*-test (^##^ *p* < 0.01, ^###^ *p* < 0.001).

Expression of inflammatory and fibrogenic genes in whole livers from EFV-treated mice differed from that of Hep3B and LX2 cells in vitro. None of the genes evaluated was significantly altered in liver samples (Figure 8D). To evaluate if this discrepancy was due to the counteraction in vivo of the differential effects of EFV on individual hepatocytes, HSCs and macrophages, we performed additional experiments in which mice were co-administered EFV and GdCl_3_. Intravenous injection of the latter compound induced selective depletion of macrophages in the liver, as demonstrated by the reduction in F4/80 expression (assessed by RT-PCR, immunostaining and WB; Figure 8A–C), and the animals’ responses to EFV were in line with those of Hep3B and LX2 cells in vitro. Specifically, EFV-administered mice exhibited an upregulation of fibrogenic (*Col1a1*, *Acta2*, *Vim*, *Timp1* and *Mmp2)* and inflammatory/injury markers (*Tnf*, *Il33* and *Nos2*) (Figure 8D). However, the changes observed in the expression of NLRP3 inflammasome components were not significant (data not shown), which ruled out their involvement in the mitigating role of macrophages in EFV-treated mice. Furthermore, EFV significantly reduced expression of the anti-inflammatory cytokine IL-10 only in macrophage-depleted animals (Figure 8D). Finally, assessment of MPO activity confirmed an enhanced inflammatory response in mice treated with EFV and GdCl_3_, including at higher doses, even without the depletion of macrophages (Figure 8E).

## 4. Discussion

**Figure 9:**
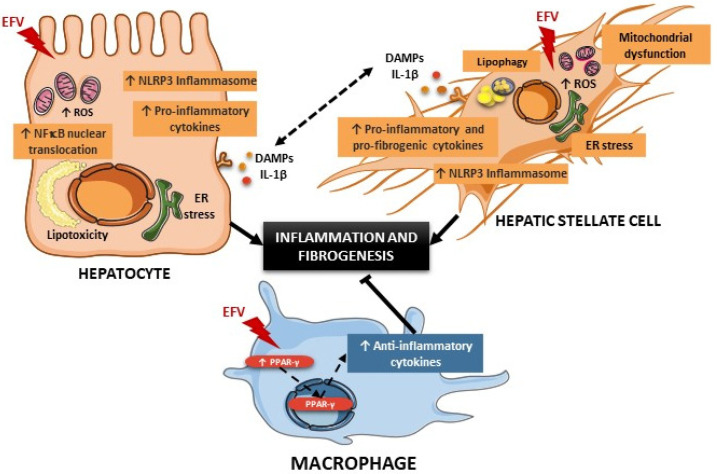
Schematic summary of the potential crosstalk between hepatocytes, hepatic stellate cells and macrophages in the liver following Efavirenz (EFV) treatment. In hepatocytes, EFV induces endoplasmic reticulum (ER) stress and mitochondrial dysfunction characterized by an enhanced reactive oxygen species (ROS) production and mitochondrial mass, and a reduction in mitochondrial membrane potential (ΔΨ*_m_*) and ATP levels, which leads to an accumulation of intracellular lipids [8,9,11]. These cellular events are associated with an increased nuclear translocation of NF-κB, which upregulates the expression of pro-inflammatory cytokines and the NLRP3 inflammasome. EFV also activates hepatic stellate cells, an effect associated with several EFV-induced actions such as enhanced oxidative stress and ER stress, and lipophagy. Moreover, EFV upregulates pro-inflammatory and fibrogenic mediators in these cells. Importantly, in both hepatocytes and hepatic stellate cells, EFV activates the NLRP3 inflammasome, which can lead to the release of damage-associated molecular patterns (DAMPs), amplifying the inflammatory response. Conversely, EFV produces macrophage polarization towards the anti-inflammatory/pro-resolving M2 phenotype, as shown by an increased expression of PPAR-γ and anti-inflammatory cytokines, which alleviates the pro-inflammatory and pro-fibrogenic response in hepatocytes and hepatic stellate cells.

The results presented in this study demonstrate cell-specific NLRP3 inflammasome activation in a pharmacological model of mitochondrial dysfunction and ER stress caused by the antiretroviral compound EFV. These effects induced acute injury in hepatocytes and HSCs, but not in macrophages (summary in Figure 9). No evidence of damage was initially found in EFV-treated animals; however, when macrophages were depleted, the detrimental effects previously detected in hepatocytes and HSCs in vitro were manifested in vivo, thus emphasizing the crucial role of macrophages in the mediation of drug-induced liver injury.

Hepatocytes activate adaptive responses to cope with the stress caused by toxic conditions, generally by producing inflammation (coupled with the recruitment of immune cells) that worsens as the insult intensifies or continues [24]. In our study, EFV induced pro-inflammatory mediators including TNF-α, overproduction of ROS, and accumulation of misfolded proteins in the ER, which resulted in the activation of NF-κB- and/or NLRP3-dependent pathways and the production of key mediators of inflammation and fibrogenesis. Particularly, EFV enhanced the production and secretion of IL-1β, a crucial feature of pathological conditions such as alcoholic liver disease and non-alcoholic fatty liver disease (NAFLD) [25,26], and induced *SERPINE1* transcription via NF-κB, a parameter associated with enhancement of fibrogenesis in NAFLD [27].

A direct role for NLRP3 in HSC activation has also been suggested [27]. In addition, these cells promote the progression of injury by regulating NF-κB and/or the NLRP3 inflammasome [28,29,30]. In line with this, our experiments demonstrate that incubation with EFV induces different molecular mechanisms involved in the activation of HSCs; namely, enhanced oxidative and ER stress, autophagy of lipid droplets, and upregulation of inflammatory and fibrogenic genes, including the NLRP3 inflammasome. Our gene array experiments show that EFV alters other important mediators of HSCs; specifically, it induces overexpression of IL-33. This cytokine is strongly associated with liver fibrosis and is produced by these cells under pro-inflammatory conditions [31]. Moreover, *P2RX7* and *PANX1* upregulation in HSCs confirms the potential of EFV to activate inflammatory pathways, either directly—by regulating mRNA expression—or indirectly—by increasing extracellular levels of ATP following mitochondrial dysfunction. Indeed, the P2RX7-mediated pathway is known to induce the NLRP3 inflammasome [32], which further endorses a NLRP3-dependent pro-inflammatory response in EFV-exposed cells. Illustrative of the challenges met when extrapolating the effects of individual cell populations to a functional liver, the unequivocal inflammatory and fibrogenic response of hepatocytes and HSCs to EFV in vitro was not reproduced in vivo. Given evidence that macrophages are essential mediators of the progression of liver injury, we investigated their role in our results. Our data show that EFV-treated macrophages exhibited an increased expression of NLRP12, a negative regulator of inflammatory responses [33,34] capable of undermining NF-κB activation in macrophages, thereby decreasing the expression of many cytokines dependent on this pathway. Furthermore, EFV induces polarization towards the M2 (or anti-inflammatory) phenotype, an effect that could be associated with increased PPARγ expression, which has previously been characterized as an anti-inflammatory mediator in liver macrophages [35,36]. This polarization towards the M2 phenotype and the activation of the abovementioned resolving pathways may explain why EFV-induced pro-inflammatory and pro-fibrogenic effects manifested in hepatocytes and HSCs do not result in evident liver injury in vivo. Our experiments in which we depleted hepatic macrophages confirm this hypothesis and attribute a fundamental role to this cell type in the control of the acute injury induced by EFV.

Mounting evidence challenges the hepatic safety profile of EFV. Besides deleterious actions in hepatocytes in vitro [7,8,9,10,11,37,38], there are clinical reports of hepatic toxicity [39,40,41,42,43] and of an association between cumulative exposure to this NNRTI and hepatic steatosis progression and hypercholesterolemia [18,44]. The present data suggest a complex scenario in which the organism is capable of counteracting the noxious profile of the drug in the majority of patients, but in which yet-to-be-defined risk factors that contribute to the development of acute or chronic liver diseases could tip the balance in specific cases. These risk factors may comprise an additional activation of inflammasomes, such as that produced by saturated fatty acids, which have been reported to induce upregulation of the inflammasome and release of danger signals in immune cells [45], or the inflammasome activation typical of the pathophysiology of alcohol- and acetaminophen-induced injury [46,47,48] circumstances that are not unusual among HIV-infected patients. Moreover, EFV-induced hypercholesterolemia and hepatic steatosis may contribute to the progression of liver injury and fibrosis in some patients. Another possible factor underlying the variable toxicological profile of EFV drawn here is the previously reported link between HIV and liver fibrosis, as the virus itself infects HSCs—promoting the expression of collagen-I and some chemokines—and regulates the progression of hepatic fibrosis through a NF-κB-dependent generation of ROS [17,49]. All these factors may be of relevance regarding the potential of this compound and other *life-long* antiretroviral drugs to modulate inflammatory and fibrotic complications, particularly as liver injury is a leading cause of death in HIV-infected patients and HIV is characterized by a sustained systemic inflammation, even when the virus is controlled [50].

## 5. Conclusions

The present study highlights a clear pro-inflammatory and pro-fibrogenic effect of EFV in hepatocytes and HSCs that is triggered by NF-κB- and NLRP3 inflammasome-dependent pathways. These responses seem to be counteracted by macrophages and may be modified by the diverse conditions typical of the clinical setting of people living with HIV. It is important to note that the potential of EFV to promote macrophage polarization towards an anti-inflammatory phenotype is a novel and unexpected property with possible therapeutic applications in the management of acute and chronic diseases. The complex response suggested by our data in this model of mitochondrial dysfunction and ER stress emphasizes the dynamic interaction among the liver’s cell populations, which requires further characterization to ascertain the molecular mechanisms that resolve inflammatory and fibrogenic responses in this organ.

## Supplementary Materials

The following supporting information can be downloaded at: https://www.mdpi.com/article/10.3390/biomedicines10010109/s1, Supplementary Material and methods, Table S1: Specific primary antibodies used for Western blot analysis, Table S2: Sequences of primers used for quantitative PCR, Figure S1: Efavirenz treatment upregulates pro-inflammatory markers in LX2 cells, Figure S2: Efavirenz treatment enhances expression of anti-inflammatory genes in U937-derived macrophages.
