# Supplementary material for: Macrophages Modulate Hepatic Injury Involving NLRP3 Inflammasome: The Example of Efavirenz
Source: Biomedicines. 2022 Jan 5;10(1):109. doi: 10.3390/biomedicines10010109 (PMC8772956; doi:10.3390/biomedicines10010109)
Supplement: Supplementary file 1 [file biomedicines-10-00109-s001.zip › biomedicines-1421902-supplementary.pdf]

SUPPLEMENTARY MATERIAL OF MANUSCRIPT ENTITLED: "Macrophages modulate hepatic injury involving NLRP3 inflammasome activation: the example of Efavirenz"

Fernando Alegre, Alberto Martí-Rodrigo, Miriam Polo, Dolores Ortiz-Masiá, Celia Bañuls, Marcello Pinti, Ángeles Álvarez, Nadezda Apostolova, Juan V. Esplugues, Ana Blas-García

Material and methods:

Cell culture and treatments. Human hepatoblastoma Hep3B cells (ATCC HB-8064) were cultured as described previously (Blas-García et al, 2010). Human immortalized HSCs LX2 (kindly provided by Dr. Scott L. Friedman, Icahn School of Medicine at Mount Sinai, New York, NY) were grown in DMEM culture medium supplemented with 10% heat-inactivated foetal bovine serum and penicillin (50U/mL) and streptomycin (50mg/mL). U937 human monocytes (European Collection of Cell Culture, Salisbury, UK) were cultured in RPMI medium supplemented with 10% heat-inactivated foetal bovine serum and penicillin (100 U/mL) and streptomycin (100mg/mL), and were differentiated into macrophages with phorbol-12-myristate-13-acetate (PMA, 48h). Key experiments were performed in human hepatocytes, Kupffer cells (both from Gibco) and HSCs (Zenbio, Durham, NC), all cultured following the manufacturer's instructions.

Experiments (24h) were generally performed with cells treated with clinically relevant (10-25μM) concentrations of EFV, but 50μM was used in key experiments. Methanol was used as a control and had no effect on any of the parameters evaluated. Other controls employed were complex I inhibitor rotenone (25μM), ER stress inductor

thapsigargin (TG, 2μM) and a pro-inflammatory stimulus “LPS cocktail” (C.LPS) composed of *E.Coli* endotoxin (LPS, 1μg/mL), IFN-γ (500U/mL) and TNF-α (20ng/mL).

Protein extraction and Western blot analysis. Total cell protein extracts were obtained as previously described [6]. To obtain nuclear extracts, cell pellets were incubated on ice for 15 min with 50 μl of lysis buffer (10 mM HEPES, pH 7.5, 2 mM $MgCl_2$, 1 mM EDTA, 1 mM EGTA, 10 mM NaCl, 1 mM DTT, 10 mM NaF, 0.1 mM Na_3VO_4, 0.2% NP40, supplemented with protease inhibitors (“Pefabloc” and “Complete Mini” protease inhibitor cocktail, both from Roche Diagnostics). Lysates were centrifuged (10 min, 4°C, 16000 g) and supernatants were considered cytosolic extracts. Pellets were sonicated for 10 min in 50 μl nuclear extraction buffer (25 mM HEPES, pH7.5, 500 mM NaCl, 1 mM DTT, 10 mM NaF, 10% Glycerol, 0,2% NP40, 5 mM $MgCl_2$, supplemented with protease inhibitors (“Pefabloc” and “Complete Mini” protease inhibitor cocktail, both from Roche Diagnostics). Nuclear lysates were centrifuged (10 min, 4°C, 16000 g) and supernatants were considered nuclear extracts. Protein content was quantified using the ‘‘BCA Protein Assay Kit’’ (Pierce). SDS-PAGE and WB were performed using standard methods (BioRad, Hercules, CA), with 50 μg of the protein extract and employing different primary antibodies (Supplementary Table 1). The secondary antibodies were peroxidase- labeled anti-rabbit IgG (PI-1000, Vector laboratories, Burlingame, CA) at 1:5000 or anti-mouse antibody (P0260, Dako Glostrup, Denmark) at 1:2000. Immunolabelling was detected using the enhanced chemiluminescent reagent ECL (Amersham, GE Healthcare, Little Chalfont, UK) or SuperSignal WestFemto (Pierce, Thermo Scientific, Rockford, IL), and was visualized with a digital luminescent image analyzer (FUJIFILM LAS 3000, Fujifilm, Barcelona, Spain). ImageQuant software v. 4.0. was used for densitometric analysis.

Real time PCR assays. The relative expression of different genes involved in inflammation and cellular stress (*AIM2*, *CASP1*, *CASP5*, *CCL7*, *CXCL1*, *CXCL2*, *HSP90AA1*, *HSP90AB1*, *HSP90B1*, *IL18*, *IL1B*, *IL33*, *IL6*, *MAP3K7*, *NAIP*, *NLRP1*,

NLRP3, *NOD2*, *P2RX7*, *PANX1*, *PTGS2*, *PYCARD*, *RIPK2*, *TNFSF11* and *TRAF6*) was analysed in LX2 cells using a pre-validated set of real time PCR assays (PrimePCR assays Bio-Rad Laboratories, Hercules, CA) directly dried in wells, following the instructions provided. The expression of two housekeeping genes (HKG), *β-actin* and *GADPH*, was also analyzed in the same plates, and *β-actin*, as the most stable gene, was chosen as HKG to normalize data. All reactions were performed in a CFX96 touch real-time PCR, and analyzed with Biorad CFX Manager 3.1 software (Bio-Rad). Data are expressed as relative-fold change with respect to untreated (vehicle) sample, set to one.

The same method was used to analyse the mRNA expression of *CASP1*, *CCL2*, *CCL7*, *HSP90B1*, *IFNB1*, *IL18*, *IL1B*, *IRAK1*, *MAPK12*, *MAPK9*, *MEFV*, *MYD88*, *NAIP*, *NLRC4*, *NLRP12*, *NLRP3*, *P2RX7*, *PANX1*, *PSTPIP1*, *PTGS2*, *PYCARD*, *RIPK2*, *TNFA*, *TNFSF14* and *TNFSF4* in macrophages. The mRNA levels of two HKG, *β-actin* and *HPRT1*, was also measured, and *HPRT1* was chosen as HKG to normalize data.

Fluorescence microscopy and static fluorescence. All treatments were performed in duplicate in 48-well plates and Hoechst 33342 (2.5μM) was added to stain nuclei. 16-25 live-cell images/well were recorded with an IX81 Olympus fluorescence microscope with "ScanR" static cytometry software version 2.03.2 (Olympus, Hamburg, Germany)

Cell proliferation/survival. Cells were treated, allowed to proliferate for 24h, and counted according to Hoechst fluorescence.

Mitochondrial membrane potential, mitochondrial mass, mitochondrial superoxide production, ER signal and lysosomal signal. Specific fluorochromes were added: 5μM TMRM ($\Delta\Psi_m$), 1μM NAO (mitochondrial mass), 2.5μM MitoSOX (superoxide production), 2.5μM ER Tracker Red (ER signal) and 0.1μM Lysotracker Red (lysosome signal).

Intracellular lipid accumulation. Once nuclei were stained, cells were incubated with 0.5μM Nile Red to stain lipid droplets.

References

Blas-García A, Apostolova N, Ballesteros D, Monleón D, Morales JM, Rocha M, *et al*. Inhibition of mitochondrial function by efavirenz increases lipid content in hepatic cells. Hepatology 2010;52:115-125.

Supplementary Table S1. Specific primary antibodies used for western blot analysis.

Antibody	Dilution
**IκB-α (sc-371, Santa Cruz Biotechnology, Heidelberg, Germany)**	1:1000
**β-Actin (A5060, Sigma-Aldrich)**	1:1000
**NF-κB (p65) (339900, Invitrogen)**	1:500
**Nucleolin (N2662, Sigma-Aldrich)**	1:2500
**Caspase 1 (2225, Cell Signaling, Danvers, MA)**	1:1000
**GRP78 (ab21685, Abcam, Cambridge, UK)**	1:1000
**CHOP (ab11419, Abcam)**	1:1000
**LC3 (L8918, Sigma-Aldrich)**	1:1000
**NLRP3 (13158, Cell Signaling)**	1:1000
**PPARγ (419300, Invitrogen)**	1:1000
**GAPDH (G9545, Sigma-Aldrich)**	1:10000

Supplementary Table S2. Sequences of primers used for quantitative PCR.

Gene	Species	Sequence
**Arginase**	human	s AGGGACAGCCACGAGGAGGG<br>as AGTTTCTCAAGCAGACCAGCCTTTC
**α-SMA**	human	s CCAAGGCCAACCGGGAGAAAATGA<br>as GCATAGAGAGACAGCACCGCCTGG
**CD86**	human	s TTGCCCAGGAACTTACAAAGGA<br>as ACCAACACAATGGAGAGGGAA
**CD206**	human	s CTTTGGACGGATGGACGAGG<br>as CAAGGAAGGGTCGGATCGTG
**COL1A1**	human	s TCTGCGACAACGGCAAGGTGT<br>as CGACGCCGGTGGTTTCTTGGT
**IL-6**	human	s CACTGGTCTTTTGGAGTTTGAGG<br>as ATTTGTGGTTGGGTCAGGGG
**iNOS**	human	s ATAATGGACCCCAGGCAAG<br>as TCAGCAAGCAGCAGAATGAG
**MMP2**	human	s CAAGTTCCCCGGCGATGTC<br>as TTCTGGTCAAGGTCACCTGTC
**MMP9**	human	s GGGCCGCTCCTACTCTGCCT<br>as TCGAGTCAGCTCGGGTCGGG
**NLRP3**	human	s CTTCTCTGATGAGGCCCAAG<br>as GCAGCAAACTGGAAAGGAAG

**pro-IL-1β**	human	s TTCGACACATGGGATAACGAGG<br>as TTTTTGCTGTGAGTCCCGGAG
**pro-IL-18**	human	s ATCGCTTCCTCTCGCAAC<br>as CCAGGTTTTCATCATCTTCAGC
**SERPINE1**	human	s CGCTGTCAAGAAGACCCACA<br>as ACCTGCTGAAACACCCTCAC
**TGF-β1**	human	s CTTCAGCTCCACAGAGAAGAACTGC<br>as CACGATCATGTTGGACAACTGCTCC
**TIMP-1**	human	s AATTCCGACCTCGTCATCAGG<br>as ATCCCCTAAGGCTTGGAACC
**TNF-α**	human	s AGCCGCATCGCCGTCTCCTA<br>as CAGCGCTGAGTCGGTCACCC
**β-actin**	human	s GGACTTCGAGCAAGAGATGG<br>as CTGTACGCCAACACAGTGCT
**α-SMA**	mouse	s GTCCCAGACATCAGGGAGTAA<br>as TCGGATACTTCAGCGTCAGGA
**Casp1**	mouse	s ACAAGGCACGGGACCTATG<br>as TCCCAGTCAGTCCTGGAAATG
**COL1A1**	mouse	s GCTCCTCTTAGGGGCCACT<br>as CCACGTCTCACCATTGGGG
**F4/80**	mouse	s TGACTCACCTTGTGGTCCTAA<br>as CTTCCCAGAATCCAGTCTTTCC
**IL-10**	mouse	s TGGACAACATACTGCTAACCGA<br>as CTGGGGCATCACTTCTACCA
**IL-33**	mouse	s TGCAGGAAAGTACAGCATTCA<br>as CGGGGAAATCTTGGAGTTGG
**iNOS**	mouse	s CGCTTGGGTCTTGTTCACTC<br>as GGTCATCTTGTATTGTTGGGCTG
**MMP2**	mouse	s CAAGTTCCCCGGCGATGTC<br>as TTCTGGTCAAGGTCACCTGTC
**NLRP3**	mouse	s ATTACCCGCCCGAGAAAGG<br>as CATGAGTGTGGCTAGATCCAAG
**pro-IL-1β**	mouse	s GAAATGCCACCTTTTGACAGTG<br>as CTGGATGCTCTCATCAGGACA
**TGF-β1**	mouse	s GCGGACTACTATGCTAAAGAGGG<br>as TCAAAAGACAGCCACTCAGG
**TIMP-1**	mouse	s CTTGGTTCCCTGGCGTACTC<br>as ACCTGATCCGTCCACAAACAG
**TNF-α**	mouse	s CCCTCACACTCAGATCATCTTCT<br>as GCTACGACGTGGGCTACA
**Vimentin**	mouse	s GCTCCTACGATTCACAGCCA<br>as CGTGTGGACGTGGTCACATA
**β-actin**	mouse	s GCCAACCGTGAAAAGATGACC<br>as GAGGCATACAGGGACAGCAC

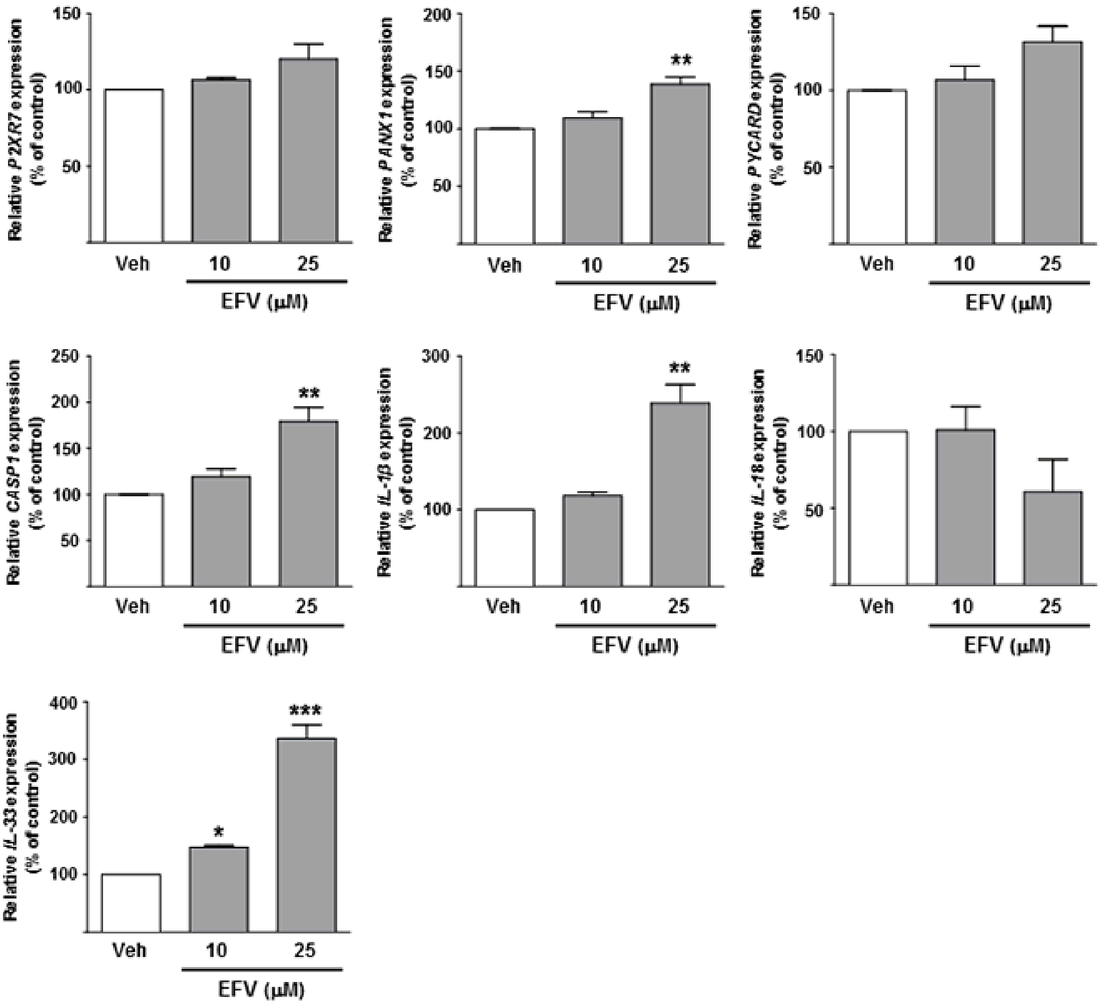

Supplementary Figure S1. Efavirenz treatment upregulates pro-inflammatory markers in LX2 cells. Relative expression of different genes involved in inflammation and cellular stress analysed using real time PCR assays. Data (mean±SEM, n=3) were calculated as percentage of control (untreated cells) and analysed by one-way ANOVA multiple comparison test followed by a Newman-Keuls test (*P< 0.05, **P<0.01, ***P<0.001 versus the respective solvent).

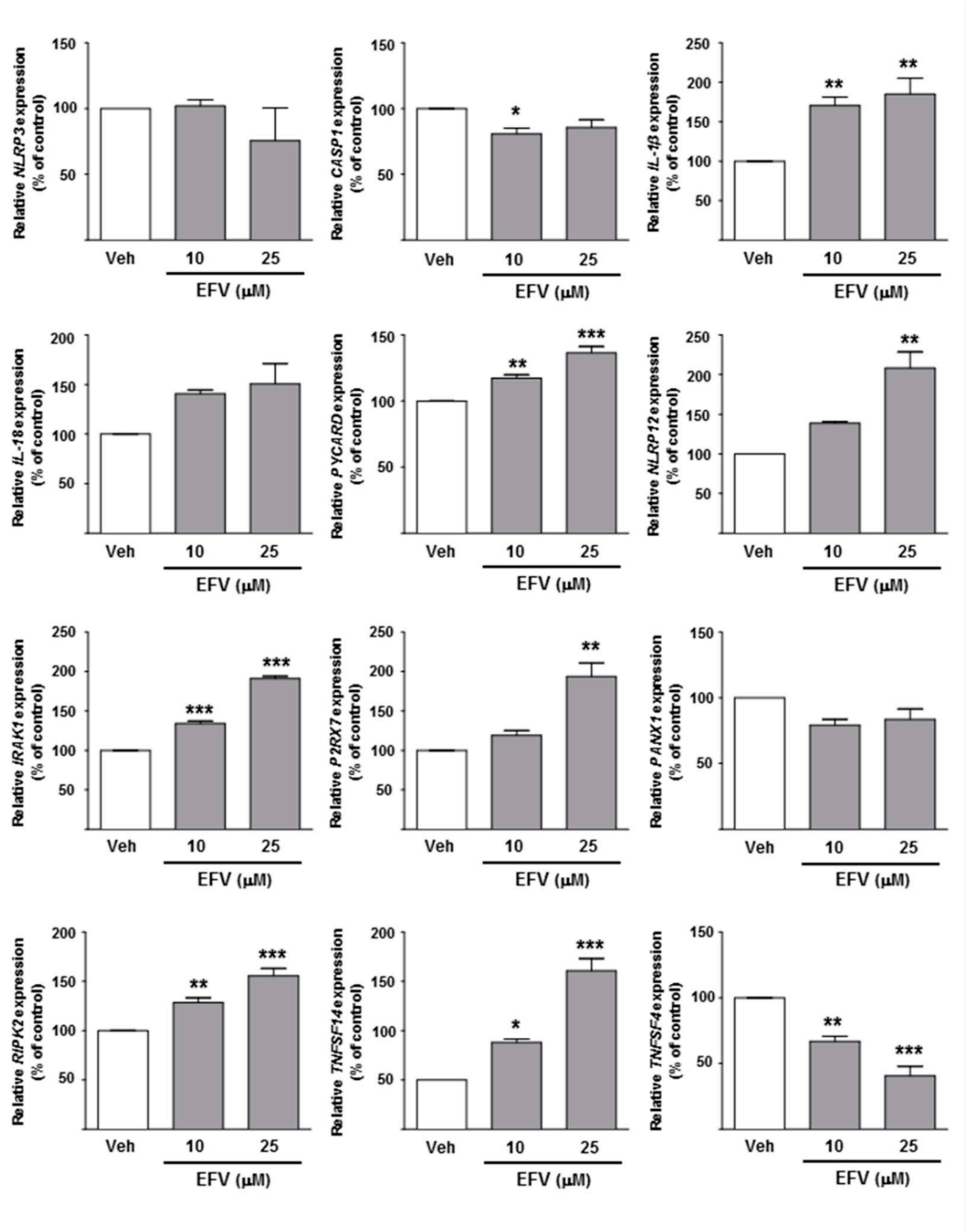

Supplementary Figure S2. Efavirenz treatment enhances expression of anti-inflammatory genes in U937-derived macrophages. Relative expression of different genes involved in inflammation and cellular stress analysed using real time PCR assays. Data (mean±SEM, n=3) were calculated as percentage of control (untreated cells) and analysed by one-way ANOVA multiple comparison test followed by a Newman-Keuls test (*P< 0.05, **P<0.01, ***P<0.001 versus the respective solvent).
